# Unmet need for family planning among adolescent girls and young women (15–24 years) in Ethiopia during the FP2020 period: a propensity score matching analysis using PMA data

**DOI:** 10.1530/RAF-26-0022

**Published:** 2026-08-12

**Authors:** Kaleb Assegid Demissie, Melak Jejaw, Getachew Teshale, Demiss Mulatu Geberu, Asebe Hagos, Nigusu Worku, Tesfahun Zemene Tafere, Endalkachew Dellie

**Affiliations:** Department of Health Systems and Policy, Institute of Public Health, College of Medicine and Health Sciences, University of Gondar, Gondar, Ethiopia

**Keywords:** FP2020, unmet need for family planning, adolescent and young women, cross-sectional pre–post design, Ethiopia

## Abstract

**Abstract:**

Adolescent and young women in Ethiopia have a high unmet need for family planning. The Family Planning 2020 strategy prioritized improving reproductive health among women aged 15–24 years. This study aims to assess the changes in the unmet need for family planning among adolescent and young women aged 15–24 years in Ethiopia during the Family Planning 2020 period. A cross-sectional pre–post study using propensity score matching analyzed two rounds of Performance Monitoring for Action data: 2014 (pre-family planning 2020) and 2021 (post-family planning 2020). Propensity score kernel matching was used, and covariate balance was assessed using standardized mean differences, median, and mean bias. A total of 3,128 adolescent and young women aged 15–24 years were included. The average treatment effect on the treated was estimated, and sensitivity analysis assessed robustness to hidden bias. The unmet need for family planning decreased from 22.9% (95% CI: 21.1–24.8) in 2014 to 17.8% (95% CI: 15.8–20.1) in 2021. Matching improved covariate balance, reducing mean bias (13.3–1.4), median bias (10.2–1.1), and standardized mean difference (58.8–5.8). The average treatment effect on the treated indicated a 2.5 percentage point reduction. Sensitivity analysis using Rosenbaum bounds showed that the effect remained significant up to Γ = 2 (*P* < 0.0001), indicating robustness to hidden bias. The unmet need for family planning among Ethiopian adolescent and young women aged 15–24 years has declined. Continued efforts under the Family Planning 2030 strategy are needed to sustain and further improve access to family planning services.

**Lay summary:**

Adolescent and young women in Ethiopia still face serious challenges in family planning. The FP2020 strategy was designed to improve the family planning experience for women between the ages of 15 and 24 years. This study seeks to evaluate the effect of the strategy on unmet needs of women for family planning by carrying out an assessment of data collected from national surveys conducted before the strategy was established in 2014 and after the establishment of the strategy in 2021. The data show that unmet needs for family planning have reduced since the establishment of the strategy. After the analysis of the variance between the study groups, the study found that there is a reduction in unmet needs for family planning. However, unmet needs remain, meaning that many young women still face challenges in obtaining contraception and making informed reproductive choices. Commitment to the new strategy, that is FP2030, is required for the improvement of family planning access and use for young women.

## Introduction

The United Nations classifies people between the ages of 15 and 24 as youth regardless of differing definitions by member states (United Nations Organisation 2013). About 1.2 billion people of this age group make up 16% of the world’s population (United Nations Organisation 2018). People between the ages of 15 and 24 exhibit distinct physical, psychological, social, and emotional changes that pose a significant risk to their lives (Ethiopia 2008, Shawon *et al.* 2023).

According to the World Health Organization, fecund and sexually active women who do not use any form of contraception and who state that they do not want any more children or would like to postpone having children are considered to have unmet needs for family planning (WHO). Many young, sexually active people worldwide who wish to avoid getting pregnant may not use contemporary FP methods for a variety of reasons, such as lack of access to services or medical professionals’ disapproval (UNFPA 2003). Youth is also a time of opportunity and risk, and their sexual choices and behaviors affect their chances for social, academic, and economic success in the future (Patton *et al.* 2016, Smith 2020).

Pregnancies among adolescents are a worldwide issue that affects nations with high, middle, and low incomes. The majority of the estimated 17 million adolescent females who give birth each year do so in low- and middle-income nations (LMICs) (Yakubu & Salisu 2018). Furthermore, there have been reports of a high rate of unwanted pregnancies among young women and teenagers in sub-Saharan Africa who are sexually active but not married (Ameyaw *et al.* 2019). Pregnancy- and delivery-related mortality is higher for young women, particularly those in their adolescent age group. Adolescent girls are thought to be responsible for 25% of the 20 million unsafe abortions and abortion-related fatalities (UNFPA 2024). Women between the ages of 15 and 24 have a higher chance of dying because the majority of pregnancies in this age range are unplanned (Calvert *et al.* 2013).

Over 30% of the 13 million adolescents and youths who have unmet needs for contraception are from sub-Saharan Africa, and only just 15% of adolescents and youths who are married or in a relationship use contemporary forms of birth control (UNFPA 2015). A trend analysis from 2000 to 2016 indicates that in Ethiopia, the prevalence of unmet need has dramatically declined over the previous 16 years (Alemu *et al.* 2024). Despite this, a higher level of unmet need among adolescent and young women was reported (Asmamaw & Negash 2023).

Ethiopia’s government is dedicated to enhancing the health of its young people. In accordance with the global strategy for women’s, children’s, and adolescents’ health (2016–2030), the nation had developed a national strategy for the health of adolescents and youths from 2016 to 2020 (PAI 2019). Within this time frame, a concerted effort will be made to increase access to contraceptives by implementing the school health program plan and expanding adolescent- and youth-friendly (AYF) clinic services (Birhanu 2014).

FP2020 is a global alliance that advocates for women’s freedom to choose whether, when, and how many children to have. To increase the number of women and girls who use contraceptives by 120 million by 2020, FP2020 collaborates with governments, civil society, multilateral organizations, donors, the commercial sector, and the research and development community (FP 2020).

As part of FP2020, the Ethiopian government pledged commitments to expand access to family planning (FP) and take appropriate action to remove some of these obstacles (Christian Aid 2022). The first commitment is to improve the health status of Ethiopian adolescents and youths by increasing contraceptive prevalence rate for women ages 15–19 years from 32 to 40% and ages 20–24 years from 38 to 43%, and reduce unmet need for contraceptive among women ages 15–19 years from 20 to 10% and among women ages 20–24 years from 18 to 10% (Ethiopia 2018). Although fulfilling FP2020 commitments could revolutionize family planning services by providing high-quality services on a large scale, reports indicate that the above and other commitments of FP2020 were lagging in Ethiopia (Christian Aid 2022). The progress in Ethiopia is hampered by the persistence of equity gaps caused by regional and/or sociodemographic differences, as well as the poor quality of FP service delivery (Kibret & Gebremedhin 2022). Therefore, the aim of this study is to assess the change in unmet need for family planning among adolescent and young women aged 15–24 years in Ethiopia across the FP2020 period, using two rounds of IPUMS-PMA datasets.

## Method

### Study design and area

We used a cross-sectional pre–post design to assess the unmet need for family planning among adolescent and young women aged 15–24 years in Ethiopia across the FP2020 period. For the purposes of this study, we combined two rounds of IPUMS-PMA datasets: the 2014 and 2021 cross-sectional datasets. We considered the 2014 dataset as a baseline and the 2021 dataset as the end line. Even though Ethiopia became a part of the FP2020 group in 2012, this strategy aimed to expand access to contraceptives by strengthening adolescent- and youth-friendly services and reducing unmet need for this age group over the four years of 2016–2020. Therefore, we take participants from round one (2014) dataset as a control group and participants from round six (2021) dataset as a treatment group to assess changes in unmet need for family planning across the FP2020 period.

### Data source

The dataset for our study was from Microdata Series – Performance Monitoring for Action (IPUMS-PMA) website (Devon Kristiansen 2024*a*,*b*). IPUMS-PMA is a collaboration between the Bill & Melinda Gates Foundation, IPUMS, and the PMA team at Johns Hopkins University. The international family planning survey series Performance Monitoring for Action (PMA; formerly known as Performance Monitoring and Accountability 2020, or PMA2020) has been standardized as IPUMS-PMA. To make pooling, trend analysis, and comparative research easier, IPUMS-PMA codes variables uniformly across nations and survey years. Users can create custom microdata files and download them for analysis using a free online web dissemination system. A definition of the variable’s meaning, a discussion of comparability concerns, sample universes (who was included in the query), and related questionnaire content are all contained in the web dissemination system’s documentation for each variable. For the purposes of our study, we used two rounds of the Ethiopian PMA cross-sectional datasets of 2014 and 2021. The data are accessible via the IPUM-PMA website https://pma.ipums.org/pma/index.shtml after providing a brief description of the study’s purpose. A total of 3,128 weighted adolescent and young women were included in the study.

### Outcome variable

Our outcome variable was a binary coded variable ‘UNMETYN’ (total unmet need) in the IPUMS-PMA dataset, which indicates a woman’s need for family planning based on her reproductive preferences, current family planning methods, and pregnancy risk factors. This binary variable is a recode of another variable ‘UNMETNEED’ constructed from the following categories: 1) unmet need for spacing, 2) unmet need for limiting, 3) met need for spacing, 4) met need for limiting, 5) not at risk, and 6) no unmet need. Women in categories 1 and 2 were defined as 1 (‘unmet need’), while women in all other categories were defined as 0 (‘no unmet need’). A further explanation for each category of ‘UNMETNEED’ is described in the IPUMS-PMA official website (Devon Kristiansen 2024*a*,*b*).

### Treatment variable

The treatment variable for our study was the variable ‘year’, so dataset prior to the implementation period were the control group and dataset after the implementation period were the treatment group. Therefore, the variable year was recoded as ‘0’ for the control group, that is, round one and ‘1’ for the treatment group, that is, round six. Variables such as age, marital status, educational status, number of other wives, region, wealth index, media exposure, health facility visits, and place of residence were considered as covariates with *P*-value < 0.05 after the chi-square test. The process of selecting covariates was based on evidence from prior research and was restricted to the availability of variables in both rounds of IPUMS-PMA dataset.

### Statistical analysis

Stata 17 was used for analysis, and adolescent and young women aged between 15 and 24 years were included in the study. The propensity score kernel matching was employed to assess changes in unmet need for family planning among adolescent and young women aged 15–24 years in Ethiopia following the FP2020 period.

To reduce potential bias in estimating the treatment effect, we applied propensity score matching (PSM) to balance observed covariates between the treatment and control groups. This method reduces selection bias in observational studies by constructing comparable groups based on similar propensity scores. PSM relies on two key assumptions: selection on observables, meaning that treatment assignment is determined by observed characteristics, and the common support condition, which ensures overlap in propensity scores between the treatment and control groups. In this study, the common support assumption was assessed using the distribution of propensity scores and the quality of covariate balance between matched groups.

The propensity score was calculated using the Stata *pscore* command. Then the *Psmatch2* command with a logistic regression estimate was employed to calculate the average treatment effect on the population (ATE), average treatment effect on the treated (ATT), and average treatment effect on the untreated (ATU). After checking different matching methods, such as radius caliper (ranging from 0.01 to 0.05), nearest neighbor with or without replacement, and kernel matching, we selected the matching technique that gives a high-quality matching using the Stata *pstest* command. The kernel matching technique was selected as this method showed lower mean bias, standardized percent bias, and pseudo-*R*^2^.

### Sensitivity analysis

Sensitivity analysis was performed to assess the robustness of our matching technique to unobservable factors since the PSM assumption of selection on observables is difficult to achieve. We used the Mantel–Haenszel (MH) test statistic to determine whether the PSM estimates were vulnerable to hidden bias because the outcome variables were binary (Rosenbaum 2002).

## Result

A total of 3,128 adolescent and young women were included in the analysis before matching, containing 1,916 (61.25%) in the control group and 1,212 (38.75%) in the treatment group. After propensity score matching, the matched sample consisted of 1,845 control and 1,165 treated group. The prevalence of unmet need among adolescent and young women was 17.8% (95% CI: 15.8–20.1) in the treatment group and 22.9% (95% CI: 21.09–24.8) in the control group. The demographic characteristics of study participants before matching were described according to the year of the survey (Table 1).

**Table 1:** Background characteristics of study participants.

Variables/category	Control	Treatment	Total
*n*	1,916	1,212	3,128
Age, years			
15–19	514	308	823
20–24	1,402	904	2,306
Current marital status			
Married	1,841	1,186	3,027
Living with partner	75	26	101
Women’s education			
No education	630	223	853
Primary	958	680	1,638
Secondary	250	230	480
Higher	80	78	158
Region			
Developed	1,811	1,113	2,924
Emerging	105	99	204
Media exposure			
No	1,209	687	1,896
Yes	707	525	1,232
Visited health facility in last year			
No	902	502	1,404
Yes	1,014	710	1,724
Husband has other wives			
No	1,785	1,156	2,941
Yes	131	56	187
Residence			
Urban	1,591	891	2,482
Rural	325	321	646
Wealth index			
Poor	830	484	1,314
Middle	383	239	622
Rich	703	489	1,192

### Propensity score estimation

**Figure 1:**
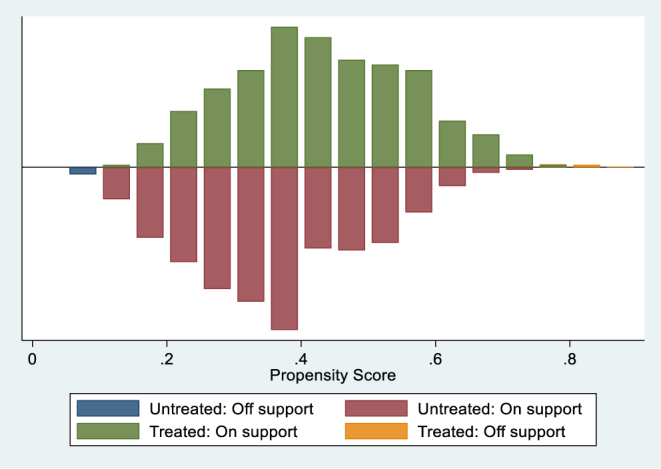
Propensity score histogram by treatment status.

**Figure 2:**
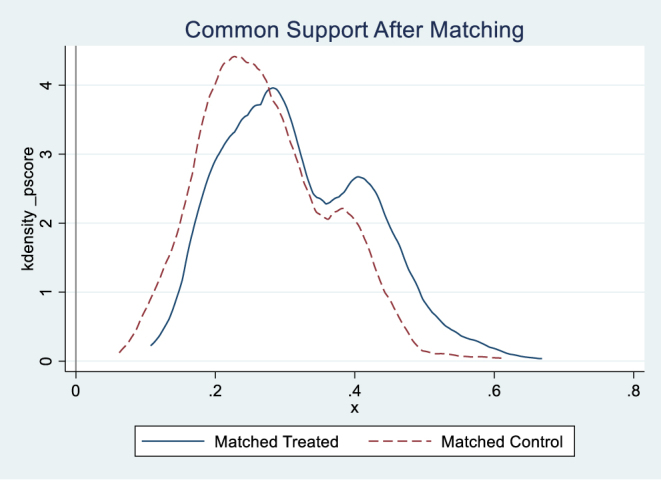
Density plot after matching.

We applied a logit estimate to calculate the propensity score of the selected covariates. The region of common support is (0.07, 0.76), as shown in Figs 1 and 2. There is a wide common support between the propensity scores of the treatment and control groups. The mean propensity score was 0.27 with a 0.11 standard deviation, and the number of blocks was 11. There were 5 off supports in the treatment group (Table 2).

**Table 2:** Common support.

	Off support	On support	Total
Treated	5	1,160	1,165
Control	14	1,831	1,845
Total	19	2,991	3,010

The average treatment effect after the FP2020 period on unmet need for family planning among adolescent and young women in Ethiopia is given in Table 3.

**Table 3:** The average treatment effect after the FP2020 period on unmet need for family planning among adolescent and young women in Ethiopia.

	Treated	Control	Difference	SE	T-stat	95% confidence interval
Unmatched	0.1639	0.1815	−0.0176	0.0142	−1.24	-
ATT	0.1637	0.1890	−0.0252	0.0151	−1.67	−0.0552209 to 0.004207
ATU	0.1807	0.1771	−0.0036	-	**-**	**-**
ATE			−0.0120	0.0152	−0.79	−0.0418746 to 0.017811

The differences in unmet need for family planning between treatment and control groups, as shown in the unmatched analysis, was 0.0176, indicating a slightly lower unmet need in the treatment group, with a standard error of 0.0142. After kernel matching, the average treatment effect on the treated (ATT) was 0.0252 suggesting that adolescent and young women who were surveyed in the post-FP2020 period had a 2.5 percentage point lower unmet need for family planning compared with their matched counterparts in the pre-FP2020 period. However, this difference was modest and did not achieve statistical significance. The average treatment effect on the untreated (ATU = 0.0036) indicated minimal change in unmet need among the control group if they had been observed in the post-FP2020 period. Similarly, the average treatment effect (ATE = 0.0120) indicated a small overall reduction in unmet need. These findings suggest a possible decline in unmet need following the FP2020 period, although the evidence does not support a definitive causal effect attributable solely to FP2020.

### Quality of matching

#### Common support

Our analysis showed that for covariates with the same propensity score, the matching technique has balanced these measured covariates between the treated group and control groups, as shown in Figs 1 and 2.

#### Balancing test, standardized bias, and model significance

**Figure 3:**
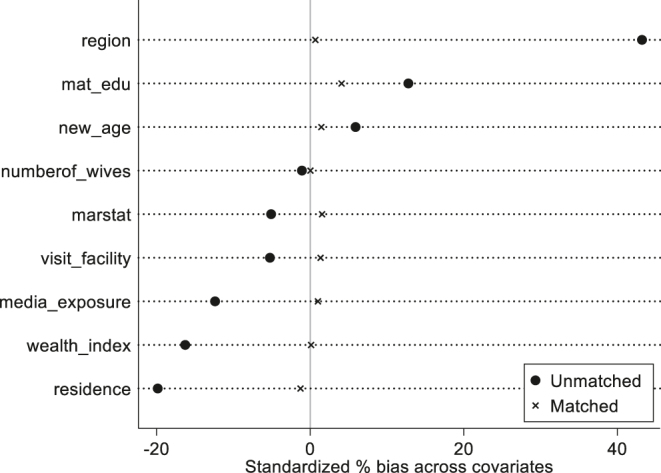
Standardized percentage bias of selected covariates.

After matching, the mean bias dropped from 13.3 to 1.4, the median bias also dropped from 10.2 to 1.1, and the standardized mean difference (B) has dropped significantly from 58.8 to 5.8. Furthermore, after matching the pseudo-*R*^2^ was 0.001 and LR chi^2^ was 1.94 with a *P*-value of 0.992, indicating that covariate distribution between the treatment and control groups is similar (Table 4). The percentage bias after matching became close to zero indicating a good-quality matching process, as illustrated in Fig. 3.

**Table 4:** Propensity score matching: quality measurements, covariate balance check, and absolute bias reduction.

Variable/sample	Mean values		% Bias	% Bias reduction	T-statistics	Samples	
	Treated	Control				Unmatched	Matched
Variables							
Age							
Unmatched	2.28	2.19	9.7		4.56		
Matched	2.28	2.27	0.9	90.9	0.35		
Marital status							
Unmatched	21.02	21.03	−4.0		−1.85		
Matched	21.02	21.92	0.2	94.6	0.09		
Wealth index							
Unmatched	1.93	2.09	−17.6		−8.21		
Matched	1.93	1.94	−1.0	94.3	−0.40		
Media exposure							
Unmatched	0.36	0.39	−6.8		−3.19		
Matched	0.36	0.36	−0.0	99.9	−0.00		
Maternal education							
Unmatched	0.75	0.66	10.7		5.06		
Matched	0.74	0.73	1.3	87.5	0.51		
Region							
Unmatched	5.40	4.57	31.7		14.8		
Matched	5.37	5.36	0.4	98.6	0.17		
Family size							
Unmatched	0.16	0.14	5.1		2.43		
Matched	0.16	0.14	−1.4	72.4	−0.55		
Residence							
Unmatched	0.27	0.33	−14.6		−6.78		
Matched	0.27	0.28	−2.7	81.3	−1.11		
Health facility visit							
Unmatched	0.53	0.54	−2.2		−1.03		
Matched	0.53	0.53	0.2	91.4	0.07		
Statistics							
Ps *R*^2^						0.059	0.001
LR chi^2^						237.72	1.94
P > chi^2^						0.000	0.992
Mean bias						13.3	1.4
Median bias						10.2	1.1
B						58.8	5.8
R						0.96	1.11

#### Sensitivity analysis

To assess the sensitivity of our findings to such hidden bias, we applied Rosenbaum’s Mantel–Haenszel bound with the assumption of no hidden bias (Γ = 1). The Mantel–Haenszel test indicated no statistically significant association between the treatment and outcome (*P* = 0.13). However, the upper-bound *P*-value became statistically significant at Γ = 1.10 (*P* = 0.018) and remained significant for all larger values of Γ = 2 (*P* = <0.001). This indicates that the observed association is insensitive to potential hidden bias (Table 5).

**Table 5:** Mantel–Haenszel bounds (mhbounds) sensitivity analysis.

Gamma	Q_mh+	Q_mh−	*P*_mh+	*P*_mh−
1	1.12	1.12	0.13	0.13
1.05	1.60	0.63	0.053	0.26
1.10	2.07	0.16	0.018	0.43
1.15	2.52	0.17	0.005	0.42
1.2	2.95	0.60	0.001	0.27
1.25	3.36	1.01	0.003	0.15
1.3	3.76	1.40	0.0008	0.07
1.35	4.14	1.78	<0.001	0.03
1.4	4.51	2.15	<0.001	0.015
1.45	4.86	2.50	<0.001	<0.001
1.5	5.21	2.84	<0.001	<0.001
1.55	5.55	3.17	<0.001	<0.001
1.6	5.87	3.49	<0.001	<0.001
1.65	6.19	3.80	<0.001	<0.001
1.7	6.49	4.11	<0.001	<0.001
1.75	6.79	4.40	<0.001	<0.001
1.8	7.09	4.69	<0.001	<0.001
1.85	7.37	4.97	<0.001	<0.001
1.9	7.65	5.24	<0.001	<0.001
1.95	7.92	5.50	<0.001	<0.001
2	8.18	5.76	<0.001	<0.001

Gamma, odds of differential assignment due to unobserved factors; Q_mh+, Mantel–Haenszel statistic (assumption: overestimation of treatment effect); Q_mh−, Mantel–Haenszel statistic (assumption: underestimation of treatment effect); *P*_mh+, significance level (assumption: overestimation of treatment effect); *P*_mh−, significance level (assumption: underestimation of treatment effect).

## Discussion

This study compares unmet need for family planning among adolescent and young women in Ethiopia between 2014 and 2021, a period during which the FP2020 strategy was implemented using a propensity score matching analysis. However, the pre–post cross-sectional design does not allow causal attribution of observed changes to FP2020. The findings should, therefore, be interpreted as temporal changes rather than direct effects of the FP2020 initiative, since the observed differences may also reflect other concurrent changes over time, including improvements in education, socioeconomic conditions, urbanization, and other health and family planning interventions implemented during this time.

Our results show a 2.5 percentage point reduction in unmet need for family planning among adolescent and young women in the period after the FP2020 strategy was implemented. This decline represents a modest improvement over time, particularly in a context where young women in Ethiopia continue to face barriers such as stigma, limited youth-friendly services, misinformation, and sociocultural sensitivities regarding contraceptive use (Jain *et al.* 2019, Titiyos *et al.* 2023). A study conducted in Ethiopia using EDHS 2016 data indicated that the prevalence of unmet needs for family planning among adolescent and young women was 28.3% (Asmamaw & Negash 2023), representing the pre-FP2020 period. In contrast, the present study shows a lower level of unmet needs in 2021. This reduction indicates an improvement over time in family planning outcomes for this age group in Ethiopia. Despite this observed reduction, the level of unmet needs remains a concern and may not fully line up with the intended FP2020 commitments (Ethiopia 2018).

Moreover, as in other countries, such as Kenya, Pakistan, India, Malawi, and Rwanda, Ethiopia emphasized youth-friendly corners in hospitals, health centers, and health posts, coupled with training in youth-friendly services (YFS) (Appleford *et al.* 2023). According to 2018 reports, YFS innovations expanded access to family planning through youth centers, technical and vocational schools, and universities. In addition, health extension workers (HEWs) broadened their contraceptive method to include implant and IUCD insertion and removal, while national guidelines and cascade training on YFS were implemented as part of the FP2020 initiative (Appleford & Emmart 2021).

Moreover, based on the report by the United Nations, in 2020, while the level of satisfied demand for family planning in most sub-Saharan African nations was below 50%, and below 25% in some, Ethiopia was among the countries that demonstrated a notable decline in unmet need for family planning over the preceding two decades (United Nations Department of Economic & Social Affairs 2020).

Furthermore, a study using Performance Monitoring for Action 2019 data reported a lower level of unmet needs for family planning in Ethiopia compared with earlier estimates from the 2016 Ethiopian Demographic and Health Survey (Central statistical agency CSA Ethiopia and ICF 2016, Gebrekidan *et al.* 2024). Similarly, studies (Gebrekidan *et al.* 2024) have documented lower levels of unmet need than those projected by the Family Planning Estimation Tool for FP2020 focus countries between 2012 and 2017 (Cahill *et al.* 2018). These findings suggest a favorable trend over time, although such changes cannot be attributed solely to FP2020, as multiple concurrent factors may also have contributed.

Despite the observed reduction in unmet need for family planning among adolescent and young women in Ethiopia during the FP2020 period, recent evidence suggests that the FP2030 commitment places even greater emphasis on adolescent and youth sexual and reproductive health (AYSRH). This includes developing national guidelines, policies, and strategies for adolescent and youth programming, clearly defining the target (Kamuyango *et al.* 2024).

Our finding is consistent with other studies reporting a decline in unmet need for family planning in Ethiopia after FP2020. This might reflect a combined effort of multiple initiatives, including Ethiopia’s Health Sector Transformation Plan I (HSTP I), which aimed to expand informed and voluntary contraceptive use to an additional 6.2 million women and to increase the modern contraceptive prevalence rate to 55% by 2020, in line with the country’s FP2020 commitments. However, given the study design, these improvements cannot be attributed solely to the FP2020 strategy (Health 2021).

### Strength and limitation

Propensity score matching improved group comparability by reducing selection bias between pre- and post-FP2020 samples, thereby enhancing the strength of observed temporal differences. Furthermore, our study focuses on unmet family planning needs, which directly aligns with FP2020 objectives, offering stakeholders useful information for evaluating and enhancing future regulations. In addition, because IPUMS-PMA datasets are standardized and thoroughly documented, data comparability and dependability are guaranteed. Despite this strength, our study also has some limitations. Only variables that are part of the model can be adjusted by PSM so results may be skewed by unmeasured variables, such as cultural views or service quality. Moreover, the 2014 survey may not represent a strict pre-FP2020 baseline, as early implementation effects may already have been present. In addition, the 2021 survey coincided with the COVID-19 pandemic, which may have influenced access to and utilization of family planning services. Furthermore, the cross-sectional nature of the study design does not allow causal attribution of observed changes to FP2020.

### Policy implication

This decline in unmet need for family planning among adolescent girls and young women between 2014 and 2021 agreed with the implementation period of the FP2020 initiative. Even though the cross-sectional nature of our study does not permit causal attribution, this temporal improvement suggests progress in family planning service utilization and access during this period. These findings support the continued prioritization of adolescent- and youth-friendly reproductive health interventions. Ethiopia may benefit from building on strategies implemented under FP2020 when advancing its commitments under FP2030.

Therefore, we recommend policymakers, legislators, and program administrators to keep encouraging their initiatives and policies while also increasing funding for adolescent and youth sexual and reproductive health (AYSRH) to satisfy the requirements and uphold the rights of adolescent and young people. Moreover, studies also indicated that unmet need still exists, especially for women of reproductive age (15–49) (Mruts *et al.* 2023). Therefore, policymakers must investigate the reasons why certain women still have difficulty obtaining contraception, with a particular emphasis on underprivileged or marginalized groups.

## Conclusion

The findings of this study indicated that the unmet need for family planning among adolescent and young women in Ethiopia declined after the FP2020 period with a 2.5 percentage point reduction observed. While these results are encouraging, the cross-sectional design does not allow for causal attribution, and the observed changes may also reflect the influence of other concurrent factors, such as socioeconomic development, improvements in education, urbanization, and other reproductive health programs. We recommend future studies to better assess the specific contribution of FP2020 to changes in the unmet need for family planning in Ethiopia using more robust causal designs.

## Declaration of interest

The authors declare that there is no conflict of interest that could be perceived as prejudicing the impartiality of the work reported.

## Funding

This research did not receive any specific grant from any funding agency in the public, commercial, or not-for-profit sector.

## Author contribution statement

KAD conceptualized the study, reviewed the literature, designed the methodology and carried out the analysis, interpreted the results, and wrote, edited, and prepared the manuscript. MJ, GT, DMG, AH, NW, and TZT were also involved in methodology, formal analysis, interpretation of the data, and writing and editing of this work. ED was involved in conceptualization, methodology, analysis, and interpretation of the results, and revised and edited the manuscript in writing. All authors have read and approved the final version of the manuscript.

## Ethical review

This research includes data only from secondary source and, therefore, ethical approval was not required. However, we had permission from IPUMS-PMA to access and use the data by providing an online request.

## Availability of data

The datasets used for this study are publicly available at IPUMS-PMA official website: https://pma.ipums.org/pma/index.shtml. According to their terms of usage, IPUMS-PMA data cannot be redistributed.
